# Cerebral Edema Associated with Ventricular Reservoirs in Two Patients: A Case Report

**DOI:** 10.1155/2012/569762

**Published:** 2012-02-26

**Authors:** Marshall C. Cress, Angela N. Spurgeon, Douglas C. Miller, N. Scott Litofsky

**Affiliations:** ^1^Division of Neurosurgery, University of Missouri, Columbia, MO 65212, USA; ^2^Department of Pathology and Anatomical Sciences, University of Missouri, Columbia, MO 65212, USA

## Abstract

Placement of ventricular reservoirs is a common practice to treat various tumors of the central nervous system (CNS). Ventricular catheter-reservoir-associated edema has been noted in the literature, but a thorough review of this literature identified no articles that examine this particular complication in neurooncology patients, specifically. We report two cases of ventricular catheter-reservoir-associated edema in patients receiving treatment for CNS metastasis.

## 1. Introduction

Placement of ventricular reservoirs for intrathecal chemotherapy is a common neurosurgical procedure that has a well-defined risk profile. The commonly described complications include infections, catheter or reservoir malfunction, hemorrhage associated with insertion of the catheter, misplacement of catheters, and tumor seeding [1]. Other, less well-elucidated risks are also described, such as porencephalic cyst development secondary to an obstructed catheter [1–5]. We present two patients who developed cerebral edema surrounding their ventricular reservoir catheters well after catheter insertion. This complication has been described once, in a child with hydrocephalus without tumor [6]. Our two adult patients who developed ventricular catheter-reservoir-associated edema did not have associated hydrocephalus.

## 2. Case Reports

The two patients were a 44-year-old male, Patient A, with adenocarcinoma of the lung, and a 42-year-old female, Patient B, with breast cancer. Both patients were diagnosed with CNS metastases after lumbar puncture yielded CSF with malignant cells found by cytologic examination. They subsequently underwent placement of right frontal Codman ventricular reservoirs with non-antibiotic-coated, silicone 8 Fr single lumen catheters (Codman and Shurtleff, Inc; Raynham, MA). Both patients later received intrathecal (IT) chemotherapy, and their CNS disease burdens were considered to be controlled clinically and radiographically.

### 2.1. Patient A

**History and Examination**

**Figure 1:**
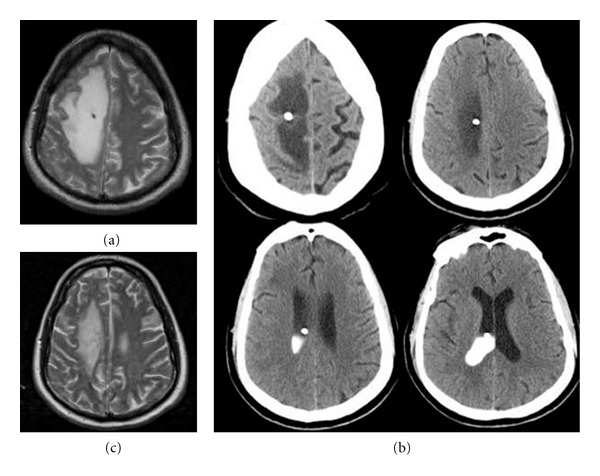
Patient A's imaging: (a) T2 MRI of the brain on admission. (b) Preoperative ventriculogram, performed through the ventricular reservoir. There is contrast within the ventricle, but it does not pass into the catheter track or the edematous white matter surrounding the catheter. (c) T2 MRI on postoperative day eight demonstrating decreased area of hyperintensity in the white matter compared to preoperative imaging.

Patient A presented 6 months after placement of his reservoir with progressive headache, nausea, vomiting, and left hemiparesis. At this time, he had received four rounds of IT cytarabine liposomal injection, the most recent two days prior to admission. An MRI of the brain on admission demonstrated new, significant edema surrounding the track of the ventricular catheter (Figure 1(a)). CSF was withdrawn easily from the reservoir and had a benign chemistry profile (Table 1). Culture and cytologic examination of the CSF were also negative for infectious agents and malignant cells. A ventriculogram performed by injecting Omnipaque into the reservoir and obtaining a CT later that day demonstrated a patent catheter without contrast extravasation into the surrounding parenchyma (Figure 1(b)). On admission, his strength was 4/5 in his left upper extremity and 3/5 in his left lower extremity. He was started on dexamethasone 4 mg q 6 hours, and subsequently had improvement in his headache, and his weakness improved to the point that he had only a mild left pronator drift. His motor function slowly began to worsen, however, despite this higher dose of steroids. Fourteen days after increasing his steroids, Patient A's motor exam had deteriorated to 1/5 in both his left arm and left leg, so the decision was made to remove his ventricular reservoir the following day.

**Operation**

The ventricular reservoir was removed and a brain biopsy was performed including cortex and underlying white matter, at the point of entry of the catheter into the brain.

**Postoperative Course**

Patient A's hemiparesis improved to 3/5 in his left arm by postoperative day three. He also had resolution of his nausea and vomiting by this time. By postoperative day ten, he no longer had headaches and his motor examination had improved to the point that he only had a left pronator drift with full strength in his left lower extremity. MRI (T2 sequence) of the brain on postoperative day eight showed improvement of the cerebral edema compared to preoperative imaging (Figures 1(a) and 1(c)). The patient was subsequently found to have widespread, intra-abdominal metastasis later in the same admission, and he died from complications related to these seven weeks after removal of his ventricular reservoir. Throughout the remainder of his hospitalization, he did not have recurrence of his neurologic findings.

### 2.2. Patient B

**History and Examination**

**Figure 2:**
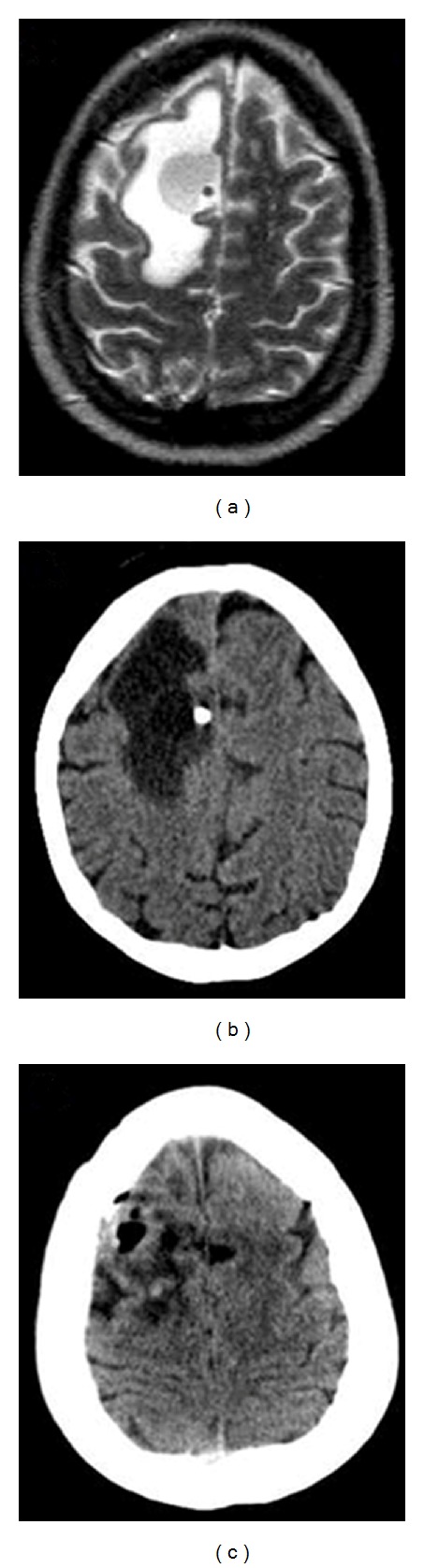
Patient B's imaging: (a) T2 MRI of the brain on admission. (b) CT of the brain on admission. (c) CT on postoperative day two, which shows resolution of the cyst and the white matter hypodensity.

Patient B presented 3 months after placement of her ventricular reservoir with headache, nausea, vomiting, diplopia, blurry vision, and left leg paresis that was causing her to fall when attempting to walk without assistance. Since placement of her ventricular reservoir, she had received three rounds of IT cytarabine liposomal injection, most recently two months prior to her presentation. On admission, Patient B's motor examination demonstrated a left pronator drift and 4/5 strength in her left lower extremity. Her admission MRI and CT of the brain showed edema surrounding the catheter track, but they also demonstrated a cyst within the edematous white matter and abutting the catheter (Figures 2(a) and 2(b)). She also had CSF withdrawn from her reservoir, which had a benign biochemical profile (Table 1) and was also negative for infectious agents and malignant cells. She was started on dexamethasone 4 mg q 6 hrs, and she was taken to the operating room the next morning.

**Operation**

Patient B also had removal of her ventricular reservoir with a simultaneous brain biopsy including cortex and underlying white matter, at the point of entry of the catheter to the brain. The cyst noted to be present preoperatively was aspirated at the time of the biopsy.

**Postoperative Course**

She had prompt resolution of her symptoms, and later on postoperative day zero, her motor examination had improved to the point that she could ambulate independently. Her left leg improved from 4/5 preoperatively to 5/5 by postoperative day one. Postoperative MRI was not performed; however, a CT of the head on postoperative day two showed significant decrease in cerebral edema and in the size of the cyst compared to her preoperative CT (Figures 2(b) and 2(c)). She was seen in clinic one week after removal of her reservoir, and she was noted to have resolution of her preoperative headache, nausea, vomiting, diplopia, blurry vision, and weakness.

## 3. Pathology

### 3.1. Patient A

**Figure 3:**
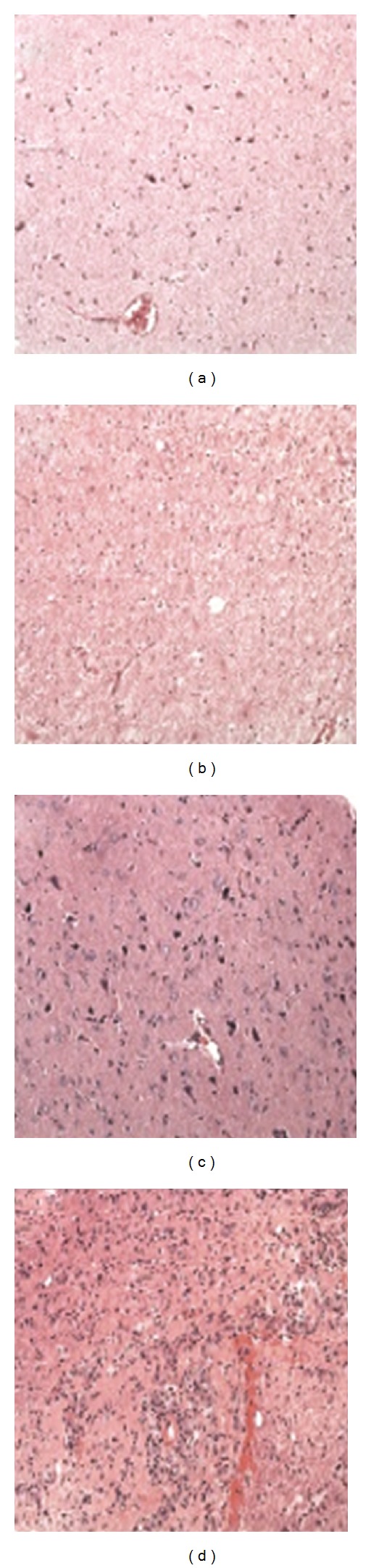
Histopathology of brain biopsies. (a) The cortex from patient A is essentially unremarkable. There is no neuronal loss or gliosis, no tumor, and no inflammation. H&E, 200x. (b) The white matter from patient A is markedly gliotic, with many large astrocytes, and is also vacuolated from edema. H&E, 200x. (c) The cortex from patient B is similarly unremarkable. H&E, 200x. (d) The white matter from patient B has perivascular infiltrates of foamy macrophages along with many large astrocytes. H&E, 200x.

As noted, the excised brain tissue included both cortex and white matter. The cortex was minimally gliotic, with no demonstrable loss of neurons and with no malignant cells or inflammatory infiltrates (Figure 3(a)). The white matter, however, was markedly gliotic with numerous large reactive astrocytes, and its background is vacuolated from edema (Figure 3(b)).

### 3.2. Patient B

The tissue excised from this patient also had both cortex and white matter, and the cortex was unremarkable (Figure 3(c)). The white matter had many large astrocytes consistent with gliosis, foci of perivascular infiltrates of foamy macrophages, and intervening early necrosis (Figure 3(d)).

## 4. Discussion

We describe two cases of cerebral edema associated with ventricular catheter reservoirs placed for intrathecal chemotherapy for malignancy. While thorough evaluation of each patient did not reveal a specific etiological mechanism, certain mechanisms can be excluded from consideration.

One possible explanation for edema formation is that the chemotherapy leaked into the brain parenchyma [7]. Prior to widespread availability of computed tomography, performing ventriculograms through a reservoir was reported as a safe way to monitor progression of brain tumors [8]. Instilling contrast through reservoirs inserted into cystic craniopharyngiomas and observing for a leak to define whether there is a potential for chemotherapeutic agents to be extruded into the brain or CSF spaces surrounding the ventricular catheter are also described [9–11]. These two techniques were adapted to evaluate patient A, and the ventriculogram demonstrated no contrast outside of the ventricles—minimizing the argument that the edema was a reaction to introduction of toxic medications into an extraventricular location. The negative ventriculogram is also contrary to the hypothesis that CSF is forced through a weak point of the ependyma associated with the point of catheter penetration [4–6].

Reports of CNS toxicity exist with the administration of IT liposomal cytarabine and also must be considered as a possible etiology. Jabbour et al. [12] reported on five patients (out of 31) with acute lymphocytic leukemia who developed CNS toxicities in the form of seizures, papilledema, cauda equine syndrome, and encephalitis after a median of four IT administrations of liposomal cytarabine via lumbar puncture. Symptoms occurred between 5–10 days after the most recent treatment, and all five patients were simultaneously receiving systemic chemotherapy for their disease. Another study by Perez-Larraya et al. [13] reported four patients (of 14) with non-Hodgkin's lymphoma, all undergoing simultaneous systemic chemotherapy, who developed either cauda equina syndrome or a pseudotumor cerebri-like syndrome. The interval between symptom development and IT treatments, as well as the median number of IT treatments before the appearance of CNS toxicity, was similar between the two studies. In contrast to the previously cited study participants, Patient B had not received any IT liposomal cytarabine for two months prior to symptom development and furthermore was not on any systemic chemotherapy at presentation. Patient A's symptoms developed two days after his most recent administration of IT liposomal cytarabine, but he was receiving only Alimta as maintenance chemotherapy for his systemic disease. Excluding the CNS toxicities related to drug administration by lumbar puncture, the development of seizure, a pseudotumor cerebri-like syndrome, and encephalitis suggest global cerebral involvement. The two patients presented in this paper displayed focal neurological deficits that were consistent with the location of the catheters thus further lending support to a focal inflammatory reaction as opposed to toxicity secondary to IT liposomal cytarabine administration.

The possibility that the edema resulted from malignant cells tracking along the catheter is unlikely because preoperative imaging (i.e., prior to removal of the catheter with brain biopsy), CSF samples, and the tissue biopsies obtained in both patients showed no sign of an active concurrent malignant process. An infectious etiology was also ruled out through the appropriate examinations of CSF from both patients, and by the absence of inflammation in the biopsies.

One important consideration is that the catheter, itself, may be the cause for the cerebral edema. Rubber ventricular catheters, subdural grids, and depth electrodes have all been reported to cause local inflammatory responses [14, 15]. Implanted silicones similar to the ventricular catheters inserted in both patients are usually well tolerated. Patients have been shown to develop antibodies to ventricular catheters comprised of this material, however [16]. A similar phenomenon has been described in maxillofacial implants [17, 18]. The finding of gliosis on pathology is also congruent with just such a process. Finally, the fact that both patients' symptoms and radiographic studies improved quickly with removal of the ventricular catheter reservoirs without subsequent recurrence is consistent with an immune reaction.

Given the findings presented, the authors suggest that development of an immune reaction must be given significant weight as a potential culprit for ventricular catheter-reservoir-associated edema. Even without an explicitly defined mechanism, however, these cases illustrate that catheter removal is necessary for resolution of the edema, symptoms, and signs. Glucocorticosteroids led to short-term but unsustained improvement, with subsequent progressive edema. Although catheter-reservoir removal may not be desirable because further intrathecal chemotherapy delivery may be compromised, such removal is necessary for prompt patient improvement.

## 5. Conclusions

These cases demonstrate that symptomatic brain edema with reactive gliosis associated with ventricular reservoirs has the potential to cause debilitating side effects in a delayed fashion months after placement of a ventricular reservoir. Cerebral edema should be discussed as a potential complication of this procedure. Prompt removal of a patient's reservoir can lead to rapid reversal of the symptomatic edema and, possibly, the associated gliosis.

## Figures and Tables

**Table 1:** Profile of the CSF withdrawn from the patients' ventricular reservoirs.

	Protein, mg/dL	Glucose, mg/dL	RBC, mcL	WBC, mcL	*N*, %	*L*, %	*M*, %
Patient A	10	106	7	0	—	—	—
Patient B	12	100	273	2	44	44	13
